# Critical Role of Klf5 in Regulating Gene Expression during Post-Eyelid Opening Maturation of Mouse Corneas

**DOI:** 10.1371/journal.pone.0044771

**Published:** 2012-09-14

**Authors:** Doreswamy Kenchegowda, Stephen A. K. Harvey, Sudha Swamynathan, Kira L. Lathrop, Shivalingappa K. Swamynathan

**Affiliations:** 1 Department of Ophthalmology, University of Pittsburgh, Pittsburgh, Pennsylvania, United States of America; 2 Department of Cell Biology and Physiology, University of Pittsburgh, Pittsburgh, Pennsylvania, United States of America; 3 McGowan Institute for Regenerative Medicine, University of Pittsburgh, Pittsburgh, Pennsylvania, United States of America; Wayne State University, United States of America

## Abstract

**Background:**

Klf5 plays an important role in maturation and maintenance of the mouse ocular surface. Here, we quantify WT and *Klf5*-conditional null (*Klf5*CN) corneal gene expression, identify Klf5-target genes and compare them with the previously identified Klf4-target genes to understand the molecular basis for non-redundant functions of Klf4 and Klf5 in the cornea.

**Methodology/Principal Findings:**

Postnatal day-11 (PN11) and PN56 WT and *Klf5*CN corneal transcriptomes were quantified by microarrays to compare gene expression in maturing WT corneas, identify Klf5-target genes, and compare corneal Klf4- and Klf5-target genes. Whole-mount corneal immunofluorescent staining was employed to examine CD45+ cell influx and neovascularization. Effect of Klf5 on expression of desmosomal components was studied by immunofluorescent staining and transient co-transfection assays. Expression of 714 and 753 genes was increased, and 299 and 210 genes decreased in PN11 and PN56 *Klf5*CN corneas, respectively, with 366 concordant increases and 72 concordant decreases. PN56 *Klf5*CN corneas shared 241 increases and 98 decreases with those previously described in *Klf4*CN corneas. Xenobiotic metabolism related pathways were enriched among genes decreased in *Klf5*CN corneas. Expression of angiogenesis and immune response-related genes was elevated, consistent with neovascularization and CD45+ cell influx in *Klf5*CN corneas. Expression of 1574 genes was increased and 1915 genes decreased in WT PN56 compared with PN11 corneas. Expression of ECM-associated genes decreased, while that of solute carrier family members increased in WT PN56 compared with PN11 corneas. Dsg1a, Dsg1b and Dsp were down-regulated in *Klf5*CN corneas and their corresponding promoter activities were stimulated by Klf5 in transient co-transfection assays.

**Conclusions/Significance:**

Differences between PN11 and PN56 corneal Klf5-target genes reveal dynamic changes in functions of Klf5 during corneal maturation. Klf5 contributes to corneal epithelial homeostasis by regulating the expression of desmosomal components. Klf4- and Klf5-target genes are largely distinct, consistent with their non-redundant roles in the mouse cornea.

## Introduction

The transparent and refractive cornea plays a central role in vision. Abnormal development and/or maintenance of the cornea result in severe defects in vision [1], [2]. Molecular and cellular events involved in corneal development, maturation and maintenance have been studied in great detail [3]–[9]. Members of different transcription factor families including Krüppel-like factors (KLF) influence corneal morphogenesis [10]–[25]. More than 17 members of the KLF family have been identified in mammals [26], [27], many of which are expressed in the ocular surface in varying amounts [17], [28], [29]. Among them, structurally related *Klf4* and *Klf5* are two of the most highly expressed transcription factors in the mouse cornea [29], [30]. Our previous studies demonstrated that both Klf4 and Klf5 are essential for normal maturation and maintenance of the mouse ocular surface [22], [31].

Klf4 and Klf5 exert tissue-dependent and non-redundant influences on the mouse ocular surface in spite of possessing an identical DNA-binding domain. Conditional disruption of *Klf4* in the developing mouse ocular surface resulted in numerous defects including corneal epithelial fragility, stromal edema, altered stromal collagen fibril organization, endothelial vacuolation and loss of mucin producing conjunctival goblet cells [21], [22], [32]. Similar conditional disruption of *Klf5* also resulted in multiple defects including translucent cornea, abnormal eyelids with malformed meibomian glands and a conjunctiva devoid of goblet cells [31]. Microarray comparison of WT and *Klf4*CN corneal and conjunctival transcriptomes identified significant differences in Klf4-target genes in these adjacent tissues, suggesting tissue-dependent functions for Klf4 [21], [33].

Here, we test the hypothesis that the basis for non-redundant functions of structurally related Klf4 and Klf5 lies in their distinct target genes in the mouse cornea. As most of the *Klf5*CN ocular surface defects appeared in post-eyelid opening stages [31], we identified the corneal Klf5-target genes before eyelid opening at PN11 and in young adults at PN56. This study design also enabled us to examine the changes in gene expression accompanying WT corneal maturation between PN11 and PN56. We report that Klf5 regulates a wide array of genes associated with a diverse spectrum of functions such as cell adhesion, barrier function, maintenance of hydration, and xenobiotic metabolism. We also show that the corneal Klf5- and Klf4-target genes are largely distinct, consistent with their non-redundant roles in the mouse cornea. Furthermore, we identified significant differences in Klf5-target genes between PN11 and PN56, revealing dynamic changes in Klf5 functions in the maturing cornea.

## Results

### Microarray analysis and validation of results

**Figure 1:**
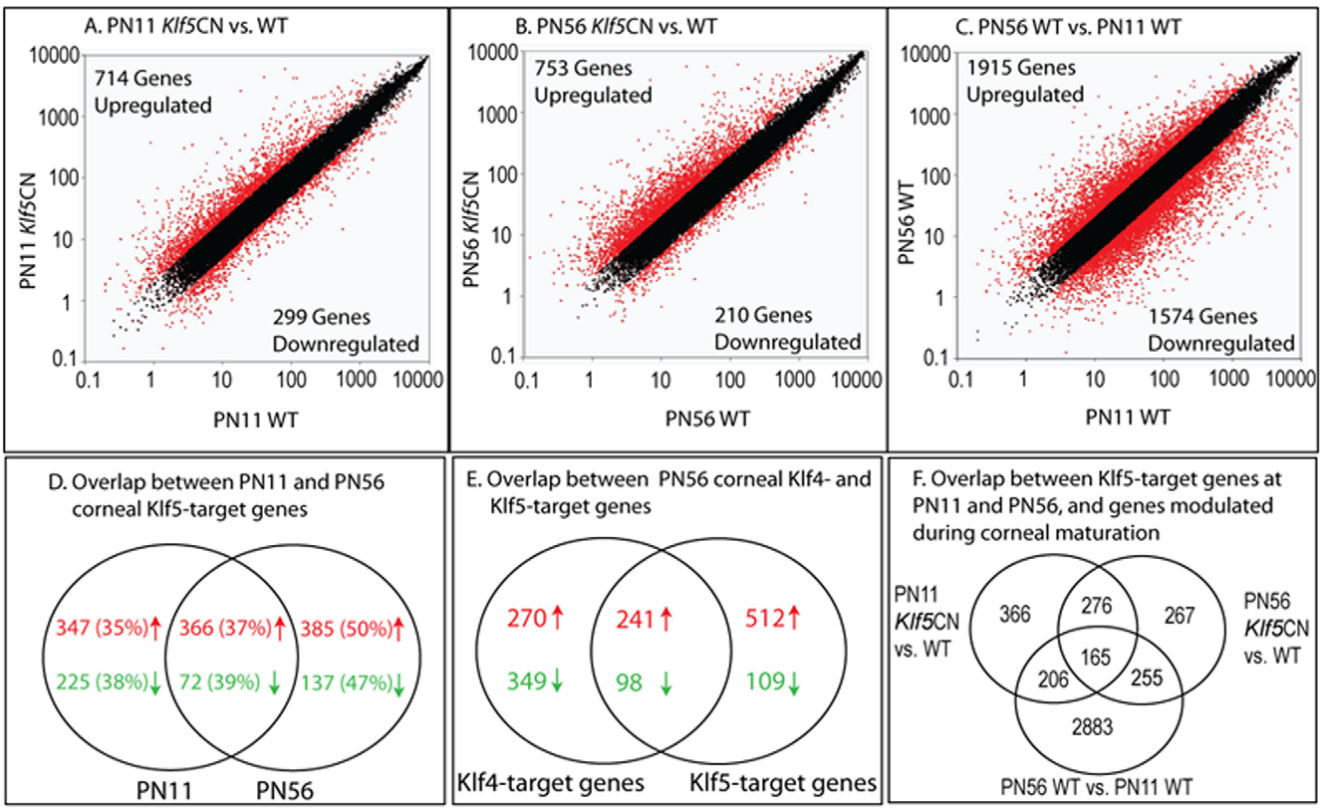
Comprehensive view of the changes in PN11 and PN56 *Klf5*CN corneal gene expression. A–C. Scatter plots showing the significantly affected genes in (A) PN11 *Klf5*CN compared with the WT corneas, (B) PN56 *Klf5*CN compared with the WT corneas, and (C) PN56 WT compared with PN11 WT corneas. (D). Venn representation of numbers of unique characterized genes which are differentially expressed in *Klf5*CN corneas vs. WT corneas at PN11 or PN56. In parentheses is the percentage of genes showing valid >2-fold changes between PN11 WT and PN56 WT samples. (E). Venn representation of the overlap between Klf4- and Klf5-target genes in PN56 corneas. (F). Venn representation of the overlap between aggregate Klf5-target genes at PN11 and PN56, and genes modulated during WT corneal maturation.

**Figure 2:**
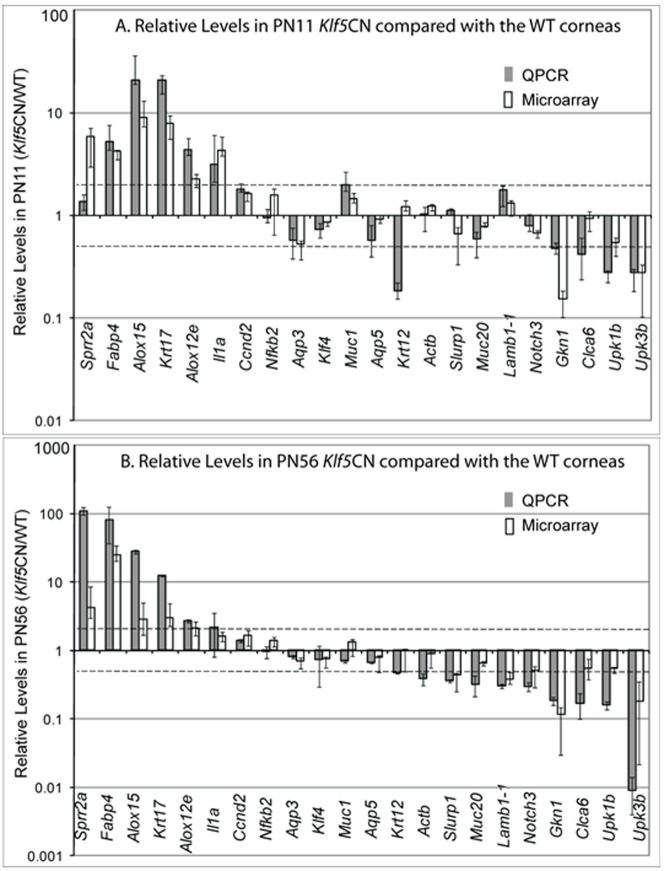
Validation of microarray results by QPCR analysis of the expression of selected genes. Note that the relative levels are plotted on a log scale.

We compared the WT and *Klf5*CN corneal transcriptomes in immature PN11 corneas just before eyelid opening and in young adult PN56 corneas to identify the changes in gene expression associated with post-eyelid opening *Klf5*CN corneal phenotype [31]. We also compared the PN56 Klf5-target genes with those reported previously for Klf4 [21] to determine the extent of overlap between Klf4- and Klf5-target genes. Scatter plots of the WT vs. *Klf5*CN comparisons at PN11 (Fig. 1A) and PN56 (Fig. 1B), and the PN11 WT vs. PN56 WT comparison (Fig. 1C) show overall distribution of the panels measured by these microarrays. A large number of genes with distinct or overlapping expression were identified between *(i)* corneal Klf5-target genes at PN11 and PN56 (Fig. 1D), *(ii)* corneal Klf4- [21] and Klf5-target genes at PN56 (Fig. 1E), and *(iii)* the genes modulated during WT corneal maturation compared with the Klf5-target genes at PN11 and PN56 (Fig. 1F). Microarray results were validated by QPCR comparison of selected genes whose expression was increased, decreased or relatively unaffected in PN11 or PN56 *Klf5*CN corneas (Fig. 2). There was a general conformity between the microarray and QPCR results, indicating that the microarray results accurately represent the changes in *Klf5*CN corneal gene expression at these two stages (Fig. 2).

### Changes in Klf5-target genes during corneal maturation

Table 1 gives a complete breakdown of the changes in 21,815 unique characterized genes represented on the microarray. Corneal ablation of *Klf5* resulted in decreased expression of 299 and 210 genes at PN11 and PN56 respectively (with 72 concordant decreases), and increased expression of 714 and 753 genes (with 366 concordant increases; Fig. 1D; Table 1). About 41% of the genes modulated in *Klf5*CN corneas were also modulated during WT corneal maturation between PN11 and PN56, compared with only 15% for those unaffected by disruption of *Klf5* (Table 1), providing evidence for the important role of Klf5 in regulating post-eyelid opening corneal maturation. The top 50 most affected genes in PN11 and PN56 *Klf5*CN corneas are listed in Tables 2, 3, 4, and 5 (See Tables S2, S3, S4, S5 for the complete list).

**Table 1:** Distribution of modulated genes.

		Developmental changes PN56 vs. PN11	PN11: Effects in *Klf5*CN vs. WT corneas		
			Increased	Decreased	Unchanged
PN56: Effects in *Klf5*CN vs. WT corneas	Increased	Increased	37 (22)	0 (0)	16 (6)
		Decreased	99 (42)	1 (0)	175 (48)
		Unchanged	230 (79)	1 (0)	194 (44)
	Decreased	Increased	0 (0)	25 (17)	47 (22)
		Decreased	0 (0)	3 (1)	17 (5)
		Unchanged	1 (0)	44 (29)	73 (26)
	Unchanged	Increased	58 (18)	31 (11)	1360 (128)
		Decreased	62 (7)	55 (7)	1503 (80)
		Unchanged	227 (16)	139 (48)	17,417 (304)

Numbers of unique characterized genes which are differentially expressed in *Klf5*CN vs. WT corneas at PN11 or at PN56 (rows) are shown. Rows are broken down according to developmental changes, i.e., differences between PN56 WT and PN11 WT corneas, as designated in the shaded column. Data sets discordant between PN11 and PN56 (i.e., increased in PN11 *Klf5*CN but decreased in PN56 *Klf5*CN, or vice versa) are too small to be meaningful. Number of genes which also show valid >2-fold changes in PN56 *Klf4*CN cornea are shown in parentheses.

**Table 2:** Top 50 genes whose expression is most decreased in PN11 *Klf5*CN compared with the WT corneas.

Gene symbol	Description	Mean log intensity in WT	Mean log intensity in *Klf5*CN	Fold Difference
Klf5	**Krüppel-like factor 5**	**9.10**	**3.87**	**0.03**
Aqp3	aquaporin 3	11.11	7.54	0.08
Folr1	**folate receptor 1 (adult)**	**8.52**	**5.01**	**0.09**
Krt4	keratin 4	7.33	4.12	0.11
Serpinb3a	serine (or cysteine) peptidase inhibitor, clade B (ovalbumin), member 3A	7.10	3.90	0.11
Ces3	**carboxylesterase 3**	**8.71**	**5.60**	**0.12**
Gkn1	**gastrokine 1**	**6.21**	**3.35**	**0.14**
Snx31	sorting nexin 31	8.59	5.84	0.15
Adh6b	alcohol dehydrogenase 6B (class V)	9.35	6.60	0.15
Ppp1r3c	**protein phosphatase 1, regulatory (inhibitor) subunit 3C**	**7.14**	**4.50**	**0.16**
Txnip	thioredoxin interacting protein	9.25	6.63	0.16
Scd2	stearoyl-Coenzyme A desaturase 2	8.88	6.27	0.16
Cyp2b19	**cytochrome P450, family 2, subfamily b, polypeptide 19**	**6.29**	**3.68**	**0.16**
Cnpy1	canopy 1 homolog (zebrafish)	5.95	3.40	0.17
Pax6	paired box gene 6	9.08	6.60	0.18
Acaa2	acetyl-Coenzyme A acyltransferase 2 (mitochondrial 3-oxoacyl-Coenzyme A thiolase)	9.39	6.94	0.18
Syt7	synaptotagmin VII	7.12	4.73	0.19
Sfrp1	secreted frizzled-related protein 1	9.60	7.21	0.19
Paqr5	**progestin and adipoQ receptor family member V**	**8.32**	**5.98**	**0.20**
Rapgef3	Rap guanine nucleotide exchange factor (GEF) 3	5.97	3.65	0.20
Fgf21	fibroblast growth factor 21	6.96	4.66	0.20
Acsm1	acyl-CoA synthetase medium-chain family member 1	8.38	6.10	0.21
Jakmip1	janus kinase and microtubule interacting protein 1	7.64	5.40	0.21
Es22	esterase 22	5.56	3.33	0.21
Gldc	**glycine decarboxylase**	**7.03**	**4.83**	**0.22**
Ripply3	ripply3 homolog (zebrafish)	6.99	4.83	0.22
Bre	brain and reproductive organ-expressed protein	9.45	7.28	0.22
Sox15	SRY-box containing gene 15	8.13	5.97	0.22
Gpt	glutamic pyruvic transaminase, soluble	8.29	6.18	0.23
Tkt	**transketolase**	**9.66**	**7.55**	**0.23**
Stk35	serine/threonine kinase 35	6.18	4.07	0.23
Ngef	neuronal guanine nucleotide exchange factor	6.49	4.39	0.23
Akr1b7	aldo-keto reductase family 1, member B7	9.73	7.63	0.23
Slc16a12	solute carrier family 16 (monocarboxylic acid transporters), member 12	8.60	6.55	0.24
Pmm1	phosphomannomutase 1	8.68	6.64	0.24
Sdc1	syndecan 1	8.01	5.99	0.25
Mamdc2	MAM domain containing 2	8.05	6.03	0.25
Ckmt1	creatine kinase, mitochondrial 1, ubiquitous	9.50	7.49	0.25
Tm9sf2	transmembrane 9 superfamily member 2	8.91	6.92	0.25
Bnc1	basonuclin 1	10.00	8.03	0.26
Cps1	carbamoyl-phosphate synthetase 1	6.29	4.34	0.26
Shmt1	serine hydroxymethyltransferase 1 (soluble)	5.82	3.88	0.26
Angptl7	angiopoietin-like 7	11.61	9.68	0.26
Ascl2	achaete-scute complex homolog 2 (Drosophila)	5.35	3.44	0.27
Padi4	peptidyl arginine deiminase, type IV	6.06	4.16	0.27
Fjx1	four jointed box 1 (Drosophila)	8.25	6.35	0.27
Slc22a18	solute carrier family 22 (organic cation transporter), member 18	6.68	4.79	0.27
Lemd1	LEM domain containing 1	5.71	3.83	0.27
Prss32	protease, serine, 32	6.95	5.11	0.28
Etv4	ets variant gene 4 (E1A enhancer binding protein, E1AF)	5.51	3.68	0.28

Genes whose expression is also decreased in the PN56 *Klf5*CN corneas are shown in bold.

**Table 3:** Top 50 genes whose expression is most increased in PN11 *Klf5*CN compared with the WT corneas.

Gene symbol	Description	Mean log intensity in WT	Mean log intensity in *Klf5*CN	Fold Difference
Sftpd	surfactant associated protein D	4.11	11.36	151.84
Ccl8	**chemokine (C-C motif) ligand 8**	**5.31**	**12.04**	**105.55**
Expi	extracellular proteinase inhibitor	6.16	12.55	83.66
Lcn2	**lipocalin 2**	**6.72**	**12.06**	**40.53**
Retnla	resistin like alpha	5.62	10.95	40.27
Ppbp	**pro-platelet basic protein**	**3.44**	**8.69**	**37.93**
Ltf	lactotransferrin	6.85	12.06	36.88
Cd209e	CD209e antigen	3.32	8.17	28.77
Cxcl3	**chemokine (C-X-C motif) ligand 3**	**3.32**	**8.13**	**27.94**
Hp	haptoglobin	3.63	8.36	26.49
S100a9	**S100 calcium binding protein A9 (calgranulin B)**	**5.64**	**10.36**	**26.43**
Ear11	eosinophil-associated, ribonuclease A family, member 11	3.32	8.01	25.81
Spink12	serine peptidase inhibitor, Kazal type 11	3.45	7.99	23.23
Cxcl17	chemokine (C-X-C motif) ligand 17	5.25	9.51	19.20
Cxcl2	chemokine (C-X-C motif) ligand 2	3.35	7.37	16.20
Cytip	cytohesin 1 interacting protein	5.55	9.54	15.84
Flt1	FMS-like tyrosine kinase 1	4.11	8.09	15.80
Cxcl5	chemokine (C-X-C motif) ligand 5	6.62	10.56	15.41
Krt16	keratin 16	6.74	10.68	15.31
Sprr1a	small proline-rich protein 1A	5.86	9.68	14.12
Nrsn1	**neurensin 1**	**3.56**	**7.24**	**12.83**
Ms4a6d	**membrane-spanning 4-domains, subfamily A, member 6D**	**5.46**	**9.07**	**12.27**
Socs3	suppressor of cytokine signaling 3	6.08	9.66	11.96
Slfn4	schlafen 4	3.87	7.43	11.75
Slco1a5	solute carrier organic anion transporter family, member 1a5	3.54	7.07	11.58
S100a8	S100 calcium binding protein A8 (calgranulin A)	7.25	10.73	11.13
Ccl9	**chemokine (C-C motif) ligand 9**	**6.52**	**9.99**	**11.11**
Spink5	serine peptidase inhibitor, Kazal type 5	5.38	8.81	10.83
Cd14	CD14 antigen	6.11	9.53	10.71
Cp	ceruloplasmin	7.60	10.98	10.40
Clec4d	**C-type lectin domain family 4, member d**	**3.72**	**7.08**	**10.26**
Stfa2l1	stefin A2 like 1	5.88	9.22	10.13
Ccl6	chemokine (C-C motif) ligand 6	5.42	8.76	10.06
Ifi203	interferon activated gene 203	5.11	8.30	9.11
Chi3l1	chitinase 3-like 1	7.81	10.98	9.00
Ccl5	chemokine (C-C motif) ligand 5	3.32	6.47	8.89
Gcnt2	glucosaminyl (N-acetyl) transferase 2, I-branching enzyme	5.21	8.32	8.65
Sprr2a	**small proline-rich protein 2A**	**3.32**	**6.43**	**8.62**
Alox15	arachidonate 15-lipoxygenase	6.68	9.74	8.34
Acpp	acid phosphatase, prostate	4.14	7.19	8.30
Ccl7	chemokine (C-C motif) ligand 7	3.65	6.70	8.30
Tm4sf1	transmembrane 4 superfamily member 1	7.39	10.43	8.22
Cxcl1	chemokine (C-X-C motif) ligand 1	5.99	9.01	8.08
Htra4	HtrA serine peptidase 4	3.32	6.33	8.02
Il1b	interleukin 1 beta	4.95	7.95	8.01
Nckap1l	NCK associated protein 1 like	3.44	6.42	7.90
Krt23	keratin 23	5.27	8.24	7.84
Plb1	phospholipase B1	4.05	7.01	7.77
Pmaip1	phorbol-12-myristate-13-acetate-induced protein 1	4.71	7.67	7.74
Slc26a4	solute carrier family 26, member 4	3.39	6.33	7.71

Genes whose expression is also increased in the PN56 *Klf5*CN corneas are shown in bold.

**Table 4:** Top 50 genes whose expression is most decreased in PN56 *Klf5*CN compared with the WT corneas.

Gene symbol	Description	Mean log intensity in WT	Mean log intensity in *Klf5*CN	Fold Difference
Klf5	**Kruppel-like factor 5**	**10.22**	**4.75**	**0.02**
Gldc	**glycine decarboxylase**	**7.88**	**4.73**	**0.11**
Ppp1r3c	**protein phosphatase 1, regulatory (inhibitor) subunit 3C**	**10.56**	**7.63**	**0.13**
Gkn1	**gastrokine 1**	**7.50**	**4.59**	**0.13**
Cyp24a1	cytochrome P450, family 24, subfamily a, polypeptide 1	7.75	5.06	0.15
Enpep	glutamyl aminopeptidase	9.38	6.72	0.16
Klk11	kallikrein related-peptidase 11	6.71	4.11	0.16
Folr1	**folate receptor 1 (adult)**	**7.79**	**5.43**	**0.20**
Cd55	CD55 antigen	6.58	4.25	0.20
Dsg1a	desmoglein 1 alpha	11.33	9.06	0.21
Trpm3	transient receptor potential cation channel, subfamily M, member 3	7.20	4.98	0.21
Il1f5	interleukin 1 family, member 5 (delta)	7.78	5.58	0.22
Pygl	liver glycogen phosphorylase	9.93	7.74	0.22
Ldlr	low density lipoprotein receptor	6.63	4.51	0.23
Lect1	leukocyte cell derived chemotaxin 1	7.83	5.72	0.23
Car3	carbonic anhydrase 3	6.11	4.01	0.23
Ces3	**carboxylesterase 3**	**10.11**	**8.04**	**0.24**
Fam184b	family with sequence similarity 184, member B	7.56	5.48	0.24
Mab21l1	mab-21-like 1 (C. elegans)	8.51	6.45	0.24
Paqr5	**progestin and adipoQ receptor family member V**	**8.08**	**6.03**	**0.24**
Dlg2	discs, large homolog 2 (Drosophila)	7.79	5.75	0.24
Sorbs2	sorbin and SH3 domain containing 2	9.45	7.41	0.24
Myo6	myosin VI	7.24	5.21	0.24
Tkt	**transketolase**	**9.65**	**7.62**	**0.25**
Slc14a1	solute carrier family 14 (urea transporter), member 1	9.34	7.32	0.25
Col4a3	collagen, type IV, alpha 3	8.91	6.89	0.25
Aldh1a1	aldehyde dehydrogenase family 1, subfamily A1	10.12	8.11	0.25
Vgll3	vestigial like 3 (Drosophila)	7.81	5.89	0.27
Abcc9	ATP-binding cassette, sub-family C (CFTR/MRP), member 9	6.78	4.86	0.27
Mmab	methylmalonic aciduria (cobalamin deficiency) type B homolog (human)	5.80	3.90	0.27
Tox	thymocyte selection-associated high mobility group box	6.12	4.24	0.27
Crygb	crystallin, gamma B	8.29	6.43	0.27
Ano4	anoctamin 4	5.57	3.71	0.28
Capsl	calcyphosine-like	6.06	4.21	0.28
Dkk1	dickkopf homolog 1 (Xenopus laevis)	7.11	5.26	0.28
Usp19	ubiquitin specific peptidase 19	6.12	4.27	0.28
Ano9	anoctamin 9	6.62	4.77	0.28
Mt3	metallothionein 3	5.17	3.32	0.28
Glb1l2	galactosidase, beta 1-like 2	5.75	3.91	0.28
Kif26a	kinesin family member 26A	6.71	4.87	0.28
Prss32	protease, serine, 32	8.46	6.67	0.29
Cyp2b19	**cytochrome P450, family 2, subfamily b, polypeptide 19**	**5.36**	**3.59**	**0.29**
Slc4a11	solute carrier family 4, sodium bicarbonate transporter-like, member 11	9.40	7.64	0.29
Cryaa	crystallin, alpha A	8.39	6.65	0.30
Scrn1	secernin 1	5.67	3.93	0.30
Phlda2	pleckstrin homology-like domain, family A, member 2	6.20	4.47	0.30
Osbpl6	oxysterol binding protein-like 6	6.50	4.77	0.30
Prkcb	protein kinase C, beta	8.48	6.77	0.31
Fxyd4	FXYD domain-containing ion transport regulator 4	7.29	5.59	0.31
Ipw	imprinted gene in the Prader-Willi syndrome region	6.07	4.38	0.31

Genes whose expression is also decreased in the PN11 *Klf5*CN corneas are shown in bold.

**Table 5:** Top 50 genes whose expression is most increased in PN56 *Klf5*CN compared with the WT corneas.

Gene symbol	Description	Mean log intensity in WT	Mean log intensity in *Klf5*CN	Fold Difference
Ppbp	**pro-platelet basic protein**	**5.28**	**10.79**	**45.32**
Sprr2d	small proline-rich protein 2D	4.73	10.14	42.29
Cxcl3	**chemokine (C-X-C motif) ligand 3**	**4.82**	**10.13**	**39.83**
Clca2	chloride channel calcium activated 2	3.32	8.40	33.76
Fabp4	fatty acid binding protein 4, adipocyte	4.37	9.44	33.53
Cd163	CD163 antigen	3.74	8.57	28.62
Sprr2f	small proline-rich protein 2F	6.65	11.49	28.60
Chi3l3	chitinase 3-like 3	3.67	8.38	26.19
Gsdmc	gasdermin C	5.18	9.70	22.88
Ccl8	**chemokine (C-C motif) ligand 8**	**6.09**	**10.59**	**22.68**
S100a8	S100 calcium binding protein A8 (calgranulin A)	6.60	11.03	21.60
Cxcl5	chemokine (C-X-C motif) ligand 5	6.93	11.10	17.96
Igj	immunoglobulin joining chain	5.48	9.64	17.95
Pip	prolactin induced protein	4.55	8.69	17.72
Ear11	eosinophil-associated, ribonuclease A family, member 11	3.50	7.64	17.65
Sprr2a	**small proline-rich protein 2A**	**7.55**	**11.69**	**17.65**
Srgn	serglycin	7.01	11.15	17.63
Saa3	serum amyloid A 3	5.00	9.04	16.45
Ms4a6d	**membrane-spanning 4-domains, subfamily A, member 6D**	**3.92**	**7.81**	**14.76**
Clca1	chloride channel calcium activated 1	3.84	7.61	13.71
Chi3l4	chitinase 3-like 4	7.05	10.80	13.50
Mrc1	mannose receptor, C type 1	5.27	9.00	13.25
Aqp4	aquaporin 4	3.99	7.70	13.03
Fcgr2b	Fc receptor, IgG, low affinity IIb	4.66	8.29	12.43
Ccl24	chemokine (C-C motif) ligand 24	3.32	6.96	12.42
Serpina3g	serine (or cysteine) peptidase inhibitor, clade A, member 3G	4.63	8.21	11.96
Nlrp10	NLR family, pyrin domain containing 10	4.43	7.98	11.74
Igl-V1	immunoglobulin lambda chain, variable 1	3.32	6.80	11.15
S100a9	**S100 calcium binding protein A9 (calgranulin B)**	**7.47**	**10.92**	**10.97**
Ccr1	chemokine (C-C motif) receptor 1	3.96	7.41	10.92
Chi3l1	chitinase 3-like 1	7.83	11.27	10.91
Csf3r	colony stimulating factor 3 receptor (granulocyte)	4.01	7.43	10.71
Il1b	interleukin 1 beta	5.71	9.11	10.56
Clec4d	**C-type lectin domain family 4, member d**	**3.84**	**7.23**	**10.51**
Mfap4	microfibrillar-associated protein 4	3.94	7.30	10.33
C1qa	complement component 1, q subcomponent, alpha polypeptide	6.22	9.58	10.25
Mmp3	matrix metallopeptidase 3	8.19	11.50	9.89
H2-Ab1	histocompatibility 2, class II antigen A, beta 1	5.92	9.20	9.72
Igh-6	immunoglobulin heavy chain 6 (heavy chain of IgM)	5.26	8.51	9.49
Clec7a	C-type lectin domain family 7, member a	5.31	8.54	9.39
Tyrobp	TYRO protein tyrosine kinase binding protein	5.16	8.37	9.25
Lcn2	**lipocalin 2**	**8.71**	**11.92**	**9.24**
Cxcl13	chemokine (C-X-C motif) ligand 13	3.85	7.04	9.12
Mmp13	matrix metallopeptidase 13	4.73	7.92	9.09
Cytip	cytohesin 1 interacting protein	5.98	9.16	9.06
Nrsn1	**neurensin 1**	**3.77**	**6.93**	**8.96**
Gatm	glycine amidinotransferase (L-arginine:glycine amidinotransferase)	4.46	7.62	8.95
S1pr3	sphingosine-1-phosphate receptor 3	4.00	7.15	8.91
Mcpt2	mast cell protease 2	3.40	6.53	8.77
Ccl9	**chemokine (C-C motif) ligand 9**	**5.93**	**9.06**	**8.74**

Genes whose expression is also increased in the PN11 *Klf5*CN corneas are shown in bold.

### Changes in gene expression during WT corneal maturation

Comparison of the WT corneal transcriptomes at PN11 and PN56 revealed that the expression of 1574 genes decreased and 1915 genes increased by more than 2-fold between PN11 and PN56 (Fig. 1C). The 50 most affected genes in the WT PN56 compared with PN11 corneas are listed in Tables 6–7 (See Tables S6 and S7 for the complete list). Transcripts encoding different collagens and other major extracellular matrix (ECM)-related proteins were significantly decreased between PN11 and PN56, suggesting that most of the ingredients for stromal ECM are produced before or around eyelid opening (Tables 7 and 8). Similarly, expression of Adam family proteinases and other MMPs that play significant roles in remodeling ECM [34] also was sharply decreased between PN11 and PN56 (Table 9), providing further evidence that most of the stromal ECM is in place by eyelid opening stage and little stromal remodeling occurs in the adult cornea [35].

**Table 6:** Top 50 genes whose expression is most increased during post-eyelid opening WT corneal maturation between PN11 and PN56.

Gene symbol	Description	Mean log intensity in PN11	Mean log intensity in PN56	Fold Difference
Dsp	**desmoplakin**	**3.56**	**10.89**	**160.99**
Lce3a	**late cornified envelope 3A**	**3.32**	**10.19**	**116.53**
Scd2	stearoyl-Coenzyme A desaturase 2	4.05	10.28	75.18
Psca	prostate stem cell antigen	6.24	11.98	53.45
Clca4	chloride channel calcium activated 4	3.44	8.65	37.16
Dsc3	**desmocollin 3**	**4.79**	**9.89**	**34.31**
Il1f9	interleukin 1 family, member 9	4.88	9.96	33.83
Lce3c	**late cornified envelope 3C**	**3.32**	**8.12**	**27.78**
Oasl1	oligoadenylate synthetase-like 1	5.47	10.26	27.52
Vps35	vacuolar protein sorting 35	3.95	8.67	26.32
Piga	phosphatidylinositol glycan anchor biosynthesis, class A	4.69	9.41	26.29
Prkci	protein kinase C, iota	4.15	8.79	25.04
Expi	extracellular proteinase inhibitor	6.16	10.76	24.20
Ggta1	glycoprotein galactosyltransferase alpha 1, 3	3.32	7.91	24.00
Il1f6	interleukin 1 family, member 6	3.91	8.45	23.34
Hif1a	hypoxia inducible factor 1, alpha subunit	3.80	8.24	21.65
Met	met proto-oncogene	3.75	8.15	21.23
Mobkl1b	MOB1, Mps One Binder kinase activator-like 1B (yeast)	4.60	9.00	21.15
Sema4d	sema domain, immunoglobulin domain (Ig), transmembrane domain (TM) and short cytoplasmic domain, (semaphorin) 4D	3.32	7.70	20.75
Pdcd6ip	programmed cell death 6 interacting protein	4.61	8.97	20.44
Mxd1	MAX dimerization protein 1	4.03	8.38	20.43
Pdlim5	PDZ and LIM domain 5	4.51	8.80	19.54
Sprr2a	**small proline-rich protein 2A**	**3.32**	**7.55**	**18.73**
Lin7c	lin-7 homolog C (C. elegans)	4.44	8.58	17.56
Sftpd	surfactant associated protein D	4.11	8.24	17.49
Adam10	a disintegrin and metallopeptidase domain 10	3.58	7.70	17.47
Vcl	Vinculin	3.32	7.44	17.42
Trp63	transformation related protein 63	3.71	7.82	17.28
Tgfbr1	transforming growth factor, beta receptor I	3.32	7.38	16.67
Slc1a1	solute carrier family 1 (neuronal/epithelial high affinity glutamate transporter, system Xag), member 1	4.61	8.54	15.26
Spink5	serine peptidase inhibitor, Kazal type 5	5.38	9.30	15.21
Sdcbp2	syndecan binding protein (syntenin) 2	4.54	8.46	15.07
Gja1	**gap junction protein, alpha 1**	**6.03**	**9.91**	**14.68**
Obfc2a	oligonucleotide/oligosaccharide-binding fold containing 2A	4.96	8.82	14.50
Cxadr	coxsackie virus and adenovirus receptor	3.75	7.61	14.50
Mff	mitochondrial fission factor	7.34	11.19	14.37
Sprr1a	**small proline-rich protein 1A**	**5.86**	**9.69**	**14.26**
Clic5	chloride intracellular channel 5	4.48	8.26	13.78
Nr1d2	nuclear receptor subfamily 1, group D, member 2	3.32	7.10	13.74
Pax6	paired box gene 6	4.54	8.30	13.60
Gch1	GTP cyclohydrolase 1	4.66	8.43	13.56
Il1a	interleukin 1 alpha	4.09	7.84	13.45
Dnm1l	dynamin 1-like	3.32	7.07	13.39
Ide	insulin degrading enzyme	4.54	8.28	13.39
Slc5a1	solute carrier family 5 (sodium/glucose cotransporter), member 1	7.41	11.14	13.28
Dock1	dedicator of cytokinesis 1	3.39	7.10	13.10
Sgpl1	sphingosine phosphate lyase 1	3.32	7.03	13.06
Malat1	metastasis associated lung adenocarcinoma transcript 1 (non-coding RNA)	7.96	11.66	13.05
Slc19a2	solute carrier family 19 (thiamine transporter), member 2	4.74	8.44	13.00
Atrx	alpha thalassemia/mental retardation syndrome X-linked homolog (human)	3.32	7.00	12.79

Genes encoding cell junctional complex components or markers of epithelial stratification are shown in bold.

**Table 7:** Top 50 genes whose expression is most decreased during post-eyelid opening WT corneal maturation between PN11 and PN56.

Gene symbol	Description	Mean log intensity in PN11	Mean log intensity in PN56	Fold Difference
Mfap4	**microfibrillar-associated protein 4**	**11.77**	**3.94**	**0.00**
Cpz	carboxypeptidase Z	10.50	3.59	0.01
Col11a1	**collagen, type XI, alpha 1**	**10.77**	**4.05**	**0.01**
Col9a1	**collagen, type IX, alpha 1**	**11.51**	**4.81**	**0.01**
Col5a2	**collagen, type V, alpha 2**	**12.06**	**5.95**	**0.01**
Meg3	maternally expressed 3	11.22	5.14	0.02
Col5a1	**collagen, type V, alpha 1**	**10.43**	**4.50**	**0.02**
Dlk1	delta-like 1 homolog (Drosophila)	10.15	4.23	0.02
Agtr2	angiotensin II receptor, type 2	10.19	4.28	0.02
Fmod	**fibromodulin**	**11.95**	**6.08**	**0.02**
Rian	RNA imprinted and accumulated in nucleus	9.47	3.61	0.02
H19	H19 fetal liver mRNA	11.32	5.48	0.02
Adamts2	**a disintegrin-like and metallopeptidase (reprolysin type) with thrombospondin type 1 motif, 2**	**9.48**	**3.68**	**0.02**
Matn4	**matrilin 4**	**11.63**	**5.95**	**0.02**
Col3a1	**collagen, type III, alpha 1**	**11.26**	**5.81**	**0.02**
Pik3cd	phosphatidylinositol 3-kinase catalytic delta polypeptide	12.06	6.73	0.03
Thbs2	**thrombospondin 2**	**11.78**	**6.49**	**0.03**
Twist2	twist homolog 2 (Drosophila)	9.03	3.82	0.03
Clec3b	**C-type lectin domain family 3, member b**	**8.77**	**3.57**	**0.03**
Aplnr	apelin receptor	9.21	4.04	0.03
Cryga	crystallin, gamma A	9.02	3.89	0.03
Mfap2	**microfibrillar-associated protein 2**	**11.15**	**6.07**	**0.03**
Fbn2	**fibrillin 2**	**9.06**	**4.00**	**0.03**
Ctsk	cathepsin K	11.39	6.39	0.03
Dpep1	dipeptidase 1 (renal)	10.50	5.53	0.03
Col6a2	**collagen, type VI, alpha 2**	**12.35**	**7.49**	**0.03**
Kazald1	Kazal-type serine peptidase inhibitor domain 1	9.89	5.05	0.04
Aspn	Aspirin	9.25	4.41	0.04
Mmp23	**matrix metallopeptidase 23**	**8.04**	**3.32**	**0.04**
***Thbs4***	**thrombospondin 4**	**11.04**	**6.35**	**0.04**
Camk4	calcium/calmodulin-dependent protein kinase IV	8.28	3.60	0.04
C1qtnf2	C1q and tumor necrosis factor related protein 2	9.40	4.80	0.04
Mcpt4	mast cell protease 4	8.04	3.44	0.04
Itih5	inter-alpha (globulin) inhibitor H5	8.06	3.47	0.04
Pi16	peptidase inhibitor 16	8.09	3.51	0.04
Lox	lysyl oxidase	11.18	6.60	0.04
Mfap5	**microfibrillar associated protein 5**	**8.13**	**3.60**	**0.04**
Col6a1	**collagen, type VI, alpha 1**	**12.49**	**7.97**	**0.04**
Col24a1	**collagen, type XXIV, alpha 1**	**7.84**	**3.32**	**0.04**
Aebp1	AE binding protein 1	9.73	5.22	0.04
Pycr1	pyrroline-5-carboxylate reductase 1	8.34	3.84	0.04
Mmp2	**matrix metallopeptidase 2**	**11.37**	**6.87**	**0.04**
Pdgfrl	platelet-derived growth factor receptor-like	11.01	6.54	0.05
B3gnt9	**UDP-GlcNAc:betaGal beta-1,3-N-acetylglucosaminyltransferase 9**	**8.44**	**3.98**	**0.05**
Tac1	tachykinin 1	8.86	4.41	0.05
Angptl7	angiopoietin-like 7	11.61	7.17	0.05
Creb3l1	cAMP responsive element binding protein 3-like 1	8.40	3.96	0.05
Cgref1	cell growth regulator with EF hand domain 1	7.74	3.32	0.05
Gpx7	glutathione peroxidase 7	9.69	5.27	0.05
Gpr124	G protein-coupled receptor 124	8.53	4.13	0.05
Igfbp4	insulin-like growth factor binding protein 4	11.62	7.24	0.05

ECM-associated genes are shown in bold.

**Table 8:** Expression of ECM-related genes between PN11 and PN56.

Gene symbol	Description	Mean log intensity for PN11	Mean log intensity for PN56	Fold Difference
**A. Collagens**				
Col11a1	collagen, type XI, alpha 1	10.77	4.05	0.01
Col9a1	collagen, type IX, alpha 1	11.51	4.81	0.01
Col5a2	collagen, type V, alpha 2	12.06	5.95	0.01
Col5a1	collagen, type V, alpha 1	10.43	4.50	0.02
Col3a1	collagen, type III, alpha 1	11.26	5.81	0.02
Col6a2	collagen, type VI, alpha 2	12.35	7.49	0.03
Col6a1	collagen, type VI, alpha 1	12.49	7.97	0.04
Col24a1	collagen, type XXIV, alpha 1	7.84	3.32	0.04
Col1a2	collagen, type I, alpha 2	13.04	8.71	0.05
Col14a1	collagen, type XIV, alpha 1	9.76	5.75	0.06
Col27a1	collagen, type XXVII, alpha 1	8.78	5.03	0.07
Col1a1	collagen, type I, alpha 1	12.83	9.08	0.08
Col2a1	collagen, type II, alpha 1	7.02	3.32	0.08
Col16a1	collagen, type XVI, alpha 1	10.92	7.52	0.10
Col11a2	collagen, type XI, alpha 2	7.33	4.04	0.10
Col6a3	collagen, type VI, alpha 3	12.48	9.36	0.12
Col5a3	collagen, type V, alpha 3	7.56	4.47	0.12
Col13a1	collagen, type XIII, alpha 1	7.10	4.11	0.13
Col15a1	collagen, type XV, alpha 1	6.78	3.85	0.13
Col20a1	collagen, type XX, alpha 1	6.01	3.32	0.16
Col4a2	collagen, type IV, alpha 2	10.76	8.37	0.19
Col8a1	collagen, type VIII, alpha 1	10.90	8.51	0.19
Col4a1	collagen, type IV, alpha 1	11.14	8.99	0.23
Col12a1	collagen, type XII, alpha 1	12.32	10.45	0.28
Col18a1	collagen, type XVIII, alpha 1	7.48	5.64	0.28
Col4a5	collagen, type IV, alpha 5	11.08	9.81	0.42
Col23a1	collagen, type XXIII, alpha 1	6.44	5.22	0.43
Col7a1	collagen, type VII, alpha 1	9.90	8.74	0.45
**B. Other ECM-related proteins**				
Lamb1-1	laminin B1 subunit 1	8.68	7.56	0.46
Lama1	laminin, alpha 1	5.30	3.32	0.25
Lama2	laminin, alpha 2	8.86	5.40	0.09
Lama4	laminin, alpha 4	7.65	3.41	0.05
Lamb2	laminin, beta 2	8.33	6.32	0.25
Lamc1	laminin, gamma 1	9.09	7.01	0.24
Lgals1	lectin, galactose binding, soluble 1	11.61	8.00	0.08
Lgals7	lectin, galactose binding, soluble 7	8.13	6.13	0.25
Lman1	lectin, mannose-binding, 1	9.95	8.89	0.48
Lum	Lumican	12.81	10.12	0.16
Sdc2	syndecan 2	7.75	6.68	0.48
Sdc3	syndecan 3	8.86	6.07	0.15
Sdc4	syndecan 4	10.70	8.21	0.18
Kera	keratocan	12.81	9.27	0.09
Vim	vimentin	11.64	8.46	0.11
Fndc1	fibronectin type III domain containing 1	9.31	6.74	0.17
Fndc3b	fibronectin type III domain containing 3B	9.45	8.13	0.40
Fndc4	fibronectin type III domain containing 4	6.29	4.22	0.24
Fndc5	fibronectin type III domain containing 5	6.34	3.69	0.16
Chad	chondroadherin	7.63	4.18	0.09
Chpf	chondroitin polymerizing factor	8.01	5.53	0.18
Chpf2	chondroitin polymerizing factor 2	8.41	6.42	0.25
Chsy3	chondroitin sulfate synthase 3	8.48	5.71	0.15
Chst1	carbohydrate (keratan sulfate Gal-6) sulfotransferase 1	5.81	4.76	0.48
Chst14	carbohydrate (N-acetylgalactosamine 4-0) sulfotransferase 14	7.87	6.29	0.33
Chst5	carbohydrate (N-acetylglucosamine 6-O) sulfotransferase 5	10.46	9.15	0.40
Chst7	carbohydrate (N-acetylglucosamino) sulfotransferase 7	6.16	3.62	0.17
Chst11	carbohydrate sulfotransferase 11	7.89	5.63	0.21
Chst12	carbohydrate sulfotransferase 12	7.30	6.03	0.41
Chst2	carbohydrate sulfotransferase 2	5.93	3.71	0.22
Has2	hyaluronan synthase 2	7.34	5.83	0.35

**Table 9:** Expression of metalloproteinases and other genes associated with matrix remodeling between PN11 and PN56.

Gene symbol	Description	Mean log intensity for PN11	Mean log intensity for PN56	Fold Difference
**A. Decreased Expression**				
Adamts2	a disintegrin-like and metallopeptidase (reprolysin type) with thrombospondin type 1 motif, 2	9.48	3.68	0.02
Adam33	a disintegrin and metallopeptidase domain 33	7.08	3.96	0.12
Adamts12	a disintegrin-like and metallopeptidase (reprolysin type) with thrombospondin type 1 motif, 12	6.55	3.52	0.12
Adam22	a disintegrin and metallopeptidase domain 22	6.96	4.46	0.18
Adamts10	a disintegrin-like and metallopeptidase (reprolysin type) with thrombospondin type 1 motif, 10	8.05	5.83	0.22
Adamts9	a disintegrin-like and metallopeptidase (reprolysin type) with thrombospondin type 1 motif, 9	6.34	4.76	0.33
Adamts3	a disintegrin-like and metallopeptidase (reprolysin type) with thrombospondin type 1 motif, 3	6.68	5.61	0.48
Mmp23	matrix metallopeptidase 23	8.04	3.32	0.04
Mmp2	matrix metallopeptidase 2	11.37	6.87	0.04
Mmp14	matrix metallopeptidase 14 (membrane-inserted)	9.59	5.52	0.06
Mmp16	matrix metallopeptidase 16	5.85	3.40	0.18
Mmp15	matrix metallopeptidase 15	7.38	5.83	0.34
Mxra8	matrix-remodeling associated 8	10.66	8.48	0.22
Mxra7	matrix-remodeling associated 7	7.65	6.15	0.35
**B. Increased Expression**				
Adam10	a disintegrin and metallopeptidase domain 10	3.58	7.70	17.47
Adam17	a disintegrin and metallopeptidase domain 17	5.07	7.41	5.06
Adamts1	a disintegrin-like and metallopeptidase (reprolysin type) with thrombospondin type 1 motif, 1	6.69	8.25	2.96

Expression of several cell-junctional complexes and late markers of stratified squamous epithelial cells increased significantly between PN11 and PN56 (shown in bold in Table 6), when much of the corneal epithelial stratification occurs. In addition, expression of several members of the solute carrier family was significantly elevated between PN11 and PN56 (Table 10), reflecting the elevated need for solute transport in metabolically active adult corneas. While specific corneal functions of many of these solute carrier family members are not known, it is noteworthy that mutations in *SLC4A11* and *SLC16A12* are associated with congenital hereditary endothelial dystrophy (CHED) [36] and microcornea [37], respectively. Another important change that takes place between PN11 and PN56 corneas is the increased expression of several oxidative stress related genes including ceruloplasmin, an antioxidant enzyme upregulated in different neurodegenerative disorders including glaucoma [38], [39], Arachidonate lipoxygenase-12 and -15, which promote epithelial wound healing and host defense [40], carbonic anhydrase-2, -12, and -13, overexpressed in human glaucoma [41]–[43], and calcium binding proteins S100A8 and A9 (Table 6), suggesting an increase in oxidative stress in the adult compared with the PN11 corneas.

**Table 10:** WT corneal expression of solute carrier family members increased between PN11 and PN56.

Gene symbol	Description	Mean log intensity in PN11	Mean log intensity in PN56	Fold Difference
Slc1a1	solute carrier family 1 (neuronal/epithelial high affinity glutamate transporter, system Xag), member 1	4.61	8.54	15.26
Slc5a1	solute carrier family 5 (sodium/glucose cotransporter), member 1	7.41	11.14	13.28
Slc19a2	solute carrier family 19 (thiamine transporter), member 2	4.74	8.44	13.00
Slc6a14	solute carrier family 6 (neurotransmitter transporter), member 14	7.93	11.48	11.70
Slc28a3	solute carrier family 28 (sodium-coupled nucleoside transporter), member 3	5.34	8.72	10.40
Slc39a8	solute carrier family 39 (metal ion transporter), member 8	4.95	8.22	9.63
Slc4a11	solute carrier family 4, sodium bicarbonate transporter-like, member 11	6.43	9.40	7.84
Slc35a3	solute carrier family 35 (UDP-N-acetylglucosamine (UDP-GlcNAc) transporter), member 3	4.36	7.02	6.31
Slc22a4	solute carrier family 22 (organic cation transporter), member 4	3.65	6.08	5.41
Slc12a2	solute carrier family 12, member 2	7.94	10.26	4.99
Slc39a6	solute carrier family 39 (metal ion transporter), member 6	5.16	7.47	4.97
Slc25a15	solute carrier family 25 (mitochondrial carrier ornithine transporter), member 15	3.62	5.76	4.42
Slc10a7	solute carrier family 10 (sodium/bile acid cotransporter family), member 7	3.48	5.60	4.35
Slc6a6	solute carrier family 6 (neurotransmitter transporter, taurine), member 6	3.32	5.35	4.09
Slc14a1	solute carrier family 14 (urea transporter), member 1	6.62	8.54	3.76
Slc25a24	solute carrier family 25 (mitochondrial carrier, phosphate carrier), member 24	6.85	8.68	3.55
Slc38a9	solute carrier family 38, member 9	4.24	5.96	3.29
Slc22a5	solute carrier family 22 (organic cation transporter), member 5	5.20	6.64	2.71
Slc25a30	solute carrier family 25, member 30	4.72	6.15	2.69
Slc11a2	solute carrier family 11 (proton-coupled divalent metal ion transporters), member 2	6.50	7.92	2.68
Slc37a1	solute carrier family 37 (glycerol-3-phosphate transporter), member 1	6.91	8.32	2.66
Slco4a1	solute carrier organic anion transporter family, member 4a1	7.36	8.75	2.61
Slc4a7	solute carrier family 4, sodium bicarbonate cotransporter, member 7	4.65	5.99	2.54
Slc44a4	solute carrier family 44, member 4	7.12	8.44	2.50
Slc38a10	solute carrier family 38, member 10	5.19	6.44	2.38
Slc5a8	solute carrier family 5 (iodide transporter), member 8	8.21	9.45	2.36
Slc20a2	solute carrier family 20, member 2	6.11	7.35	2.36
Slc44a2	solute carrier family 44, member 2	6.08	7.31	2.34
Slc25a36	solute carrier family 25, member 36	6.30	7.51	2.32
Slc38a2	solute carrier family 38, member 2	8.38	9.57	2.27
Slc4a4	solute carrier family 4 (anion exchanger), member 4	6.91	8.09	2.27
Slc16a12	solute carrier family 16 (monocarboxylic acid transporters), member 12	8.60	9.76	2.24
Slc25a13	solute carrier family 25 (mitochondrial carrier, adenine nucleotide translocator), member 13	7.49	8.62	2.20
Slc35b4	solute carrier family 35, member B4	5.66	6.79	2.19
Slc6a20a	solute carrier family 6 (neurotransmitter transporter), member 20A	5.95	7.02	2.10

### Differences in PN56 corneal Klf4- and Klf5-target genes

Comparison of the PN56 corneal Klf5-target genes with those described previously for Klf4 [21] identified 260 common targets (204 increased and 56 decreased; Fig. 1E; Tables S8 and S9), with many more modulated exclusively in the *Klf4*CN (270 increased and 349 decreased) or *Klf5*CN (512 increased and 109 decreased) corneas. Most of the common increases in *Klf4*CN and *Klf5*CN corneas are associated with immune response, reflecting enhanced inflammatory conditions in those corneas. Regulation of largely distinct sets of target genes by Klf4 and Klf5 is consistent with their non-redundant functions in the mouse cornea [22], [31].

### Elevated immune response in *Klf5*CN corneas

Canonical pathway analysis of the aggregate Klf5-target genes identified 26 significantly (p<0.001) enriched pathways, predominantly associated with immune function (Table S10). Expression of most of the genes associated with these pathways was increased, increasing the likelihood that these pathways represent indirect response to disruption of *Klf5*. To overcome this limitation, we repeated pathway analyses selectively for the genes with decreased expression in *Klf5*CN corneas. Several xenobiotic stress response-related pathways were predominantly enriched in genes with decreased expression upon disruption of *Klf5*, suggesting that Klf5 plays a role in xenobiotic stress response in the cornea (Table 11).

**Table 11:** Canonical pathways enriched among genes with reduced expression in *Klf5*CN corneas.

	PN11 *Klf5*CN		PN56 *Klf5*CN		Aggregate	
Canonical Pathway (Total number of genes in pathway)	−log(p)	Number of genes	−log(p)	Number of genes	−log(p)	Number of genes
LPS/IL-1 Mediated Inhibition of RXR Function (223)	4.5	11	3.62	8	5.67	15
Metabolism of Xenobiotics by Cytochrome P450 (197)	4.82	8	2.16	4	5.38	10
Glutathione Metabolism (92)	4.29	6		2	4.35	7
Xenobiotic Metabolism Signaling (296)	2.33	9	4.2	10	3.68	14
Arachidonic Acid Metabolism (208)	4.02	8		2	2.97	8
Fatty Acid Metabolism (185)	3.31	7		3	3.75	9
Glycerolipid Metabolism (154)	2.21	5		3	3.58	8
Mechanisms of Viral Exit from Host Cells (45)	2.84	4		1	3.18	5
Butanoate Metabolism (129)		3		3	3.08	6
Eicosanoid Signaling (77)	3.05	5		1	2.36	5
Valine, Leucine and Isoleucine Degradation (108)		3		3	2.82	6
PXR/RXR Activation (89)		2	2.78	4	2.36	5
Cysteine Metabolism (90)	2.6	4		2	2.04	4
Retinol Metabolism (61)		1	2.56	3	2.56	4
Propanoate Metabolism (122)		2		3	2.36	5
NRF2-mediated Oxidative Stress Response (192)		6		5	2.27	9
Chondroitin Sulfate Biosynthesis (68)		3	2.19	3	2.08	4
Aryl Hydrocarbon Receptor Signaling (159)		4	2.18	5		7
Urea Cycle and Metabolism of Amino Groups (78)	2.14	3				3
β-alanine Metabolism (93)		1	2.11	3		4

Pathways were selected where at least one group was significantly enriched at p<0.01 (i.e., −log(p)>2). For clarity, −log(p) cells are left blank if −log(p)<2.

**Figure 3:**
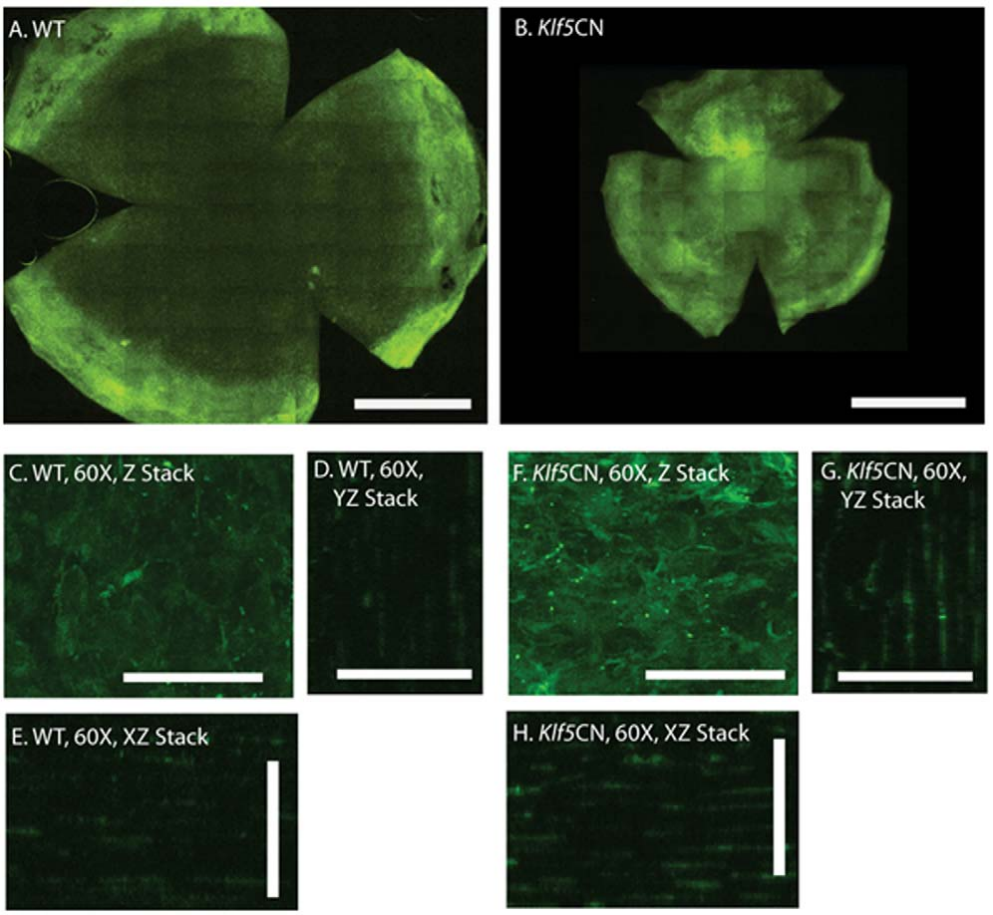
Enhanced influx of CD45+ cells into *Klf5*CN corneas. Flat mounts of PN56 WT (A) and *Klf5*CN (B) corneas were stained with FITC-conjugated anti-CD45 antibody and examined by confocal microscopy. Representative stacked images of the central corneal stroma are shown at 60× magnification (Panels C–H). Compared with the WT stroma, enhanced influx of clusters of CD45+ cells is observed throughout the depth of *Klf5*CN stromas. Scale bars: 1 mm in Panels A and B; 40 µm in Panels C–H. Data are representative of 4 independent experiments. *Klf5*CN corneas are smaller than the WT, consistent with their small eye size reported previously.

Expression of 107 of 368 genes (29.1%) containing “immun” or “inflamm” in the GO Biological Process notation was increased, while expression of only seven of these genes decreased in PN11 or PN56 *Klf5*CN corneas (Table S11). Increased expression of the immune response related genes, together with the previously reported hypercellularity of the *Klf5*CN corneal stroma [31] suggested a robust increase in immune-response in the *Klf5*CN corneas. Immunofluorescent staining of corneal flat mounts with anti-CD45 antibody demonstrated increased influx of CD45+ cells distributed throughout the *Klf5*CN compared with the WT corneal stroma sparsely populated with CD45+ cells (Fig. 3).

### *Klf5*CN corneal neovascularization (CNV)

**Figure 4:**
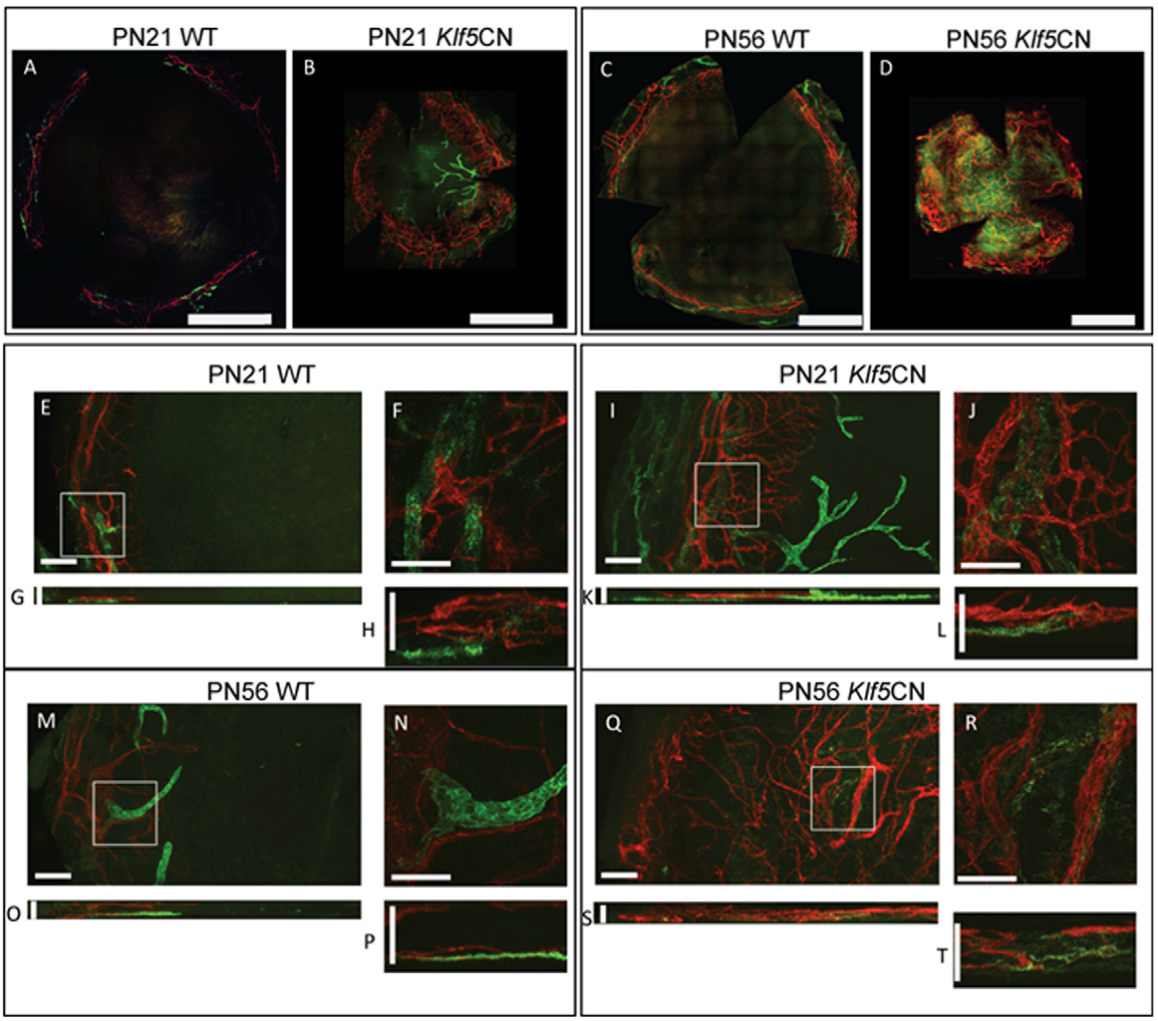
Neovascularization in *Klf5*CN corneas. Flat mounts of PN21 and PN56 WT and *Klf5*CN corneas were subjected to immunofluorescent staining with anti-CD31 (red) and anti-Lyve1 (green) antibody to detect blood vessels and lymph vessels, respectively. A–D, images of whole corneas generated by stitching together individual images from adjacent areas (Panels A–D; Scale bars = 1 mm). Vessels were blocked from entering the corneas at the limbus in WT but not *Klf5*CN corneas. Z-stack and XY-stacks of confocal images collected at 20× (panels E, G, I, K, M, O, Q and S; Scale bars = 100 µm) and 60× magnification (panels F, H, J, L, N, P, R and T; Scale bars = 50 µm) are shown. Data are representative of 4 independent experiments. *Klf5*CN corneas are smaller than the WT, consistent with their small eye size reported previously.

Whole-mount corneal immunofluorescent staining with anti-CD31 and anti-Lyve1 antibody revealed that the enhanced inflammatory environment in *Klf5*CN corneas is accompanied by extensive CNV (Fig. 4). *Klf5*CN CNV was apparent as early as PN21, when the Lyve1+ lymph vessels were much more pronounced and penetrated deeper into the central cornea, unlike the CD31+ blood vessels that remained in the peripheral region without reaching the central cornea (Fig. 4). By PN56, CNV was observed throughout the *Klf5*CN cornea, with blood vessels overtaking lymph vessels, which appeared to have regressed (Fig. 4). Examination of the XY-stack of confocal images revealed that CD31+ blood vessels are present in the anterior of the *Klf5*CN corneal stroma, unlike the Lyve1+ lymph vessels located in the posterior (Fig. 4).

### Klf5 regulates the expression of desmosomal components Dsg1a, Dsg1b and Dsp

**Figure 5:**
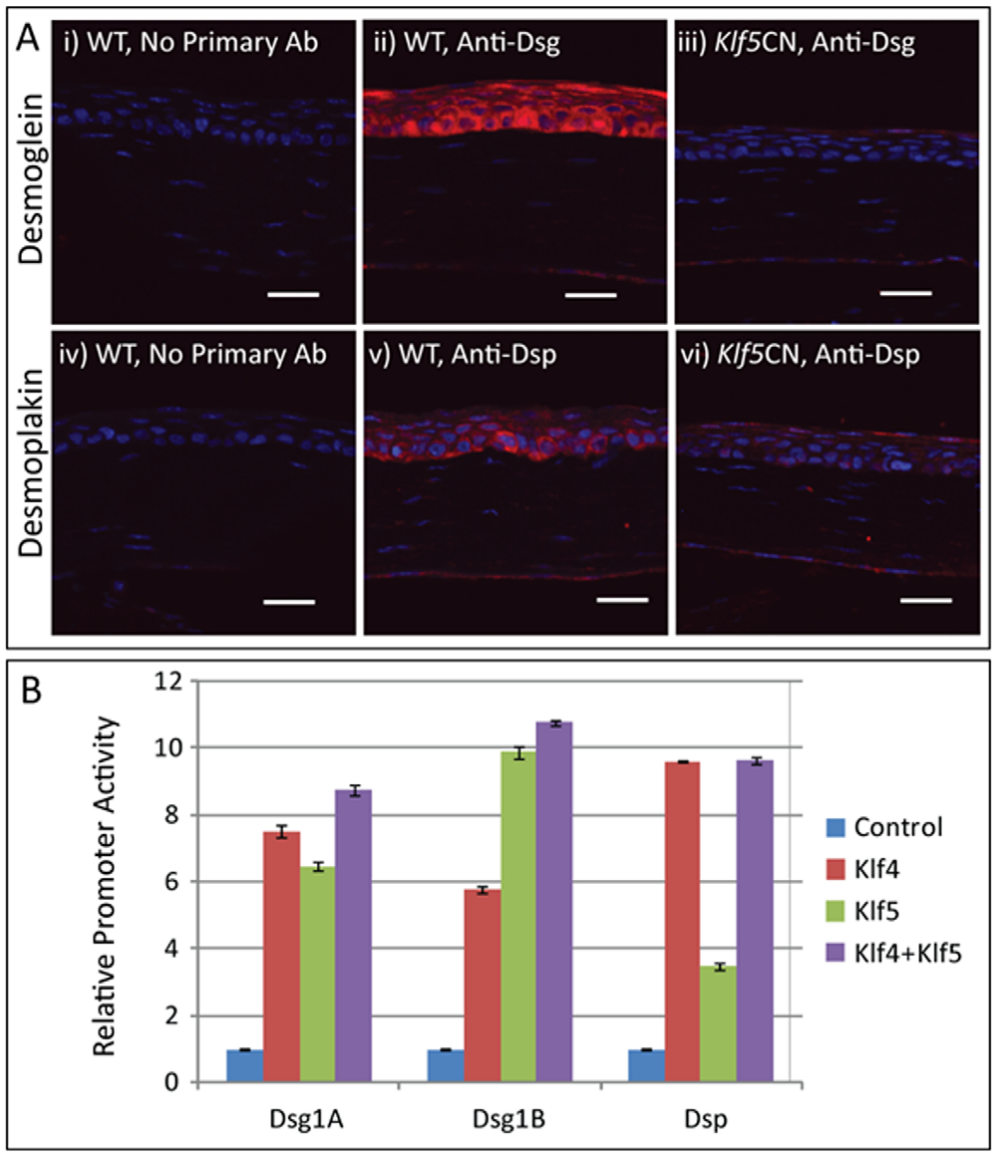
Klf5 contributes to corneal epithelial homeostasis by regulating the expression of desmogleins and desmoplakin. (A) Immunofluorescence shows abundant expression of desmogleins (red, panel ii) and desmoplakin (red, panel v) in the WT but not in the *Klf5*CN corneal epithelium (panels iii and vi, respectively). Sections processed in a similar manner but without the primary antibody served as negative controls (panels i and iv). Scale bars = 25 µm. (B) Relative activities of 2 kb *Dsg1a*, *Dsg1b* and *Dsp* promoter fragments measured by transient co-transfection assays with or without Klf4, Klf5, or both in NCTC cells. Error bars indicate Standard Error of Mean (SEM). Data are representative of three independent experiments.

Desmosomes are essential for corneal epithelial homeostasis [44]–[46]. Previously, we reported that Klf4 contributes to the formation and maintenance of corneal epithelial permeability barrier by regulating the expression of desmosomal components [47]. Microarray data presented here revealed that desmosomal components *Dsg1a* and *Dsg1b* are decreased in *Klf5*CN corneas (Table 4, Tables S2 and S4). Consistent with the microarray data, immunofluorescence revealed a sharp decrease in the epithelial expression of desmogleins, and a moderate decrease in desmoplakin in the *Klf5*CN corneas (Fig. 5A). Next, we tested the effect of Klf4 and/or Klf5 on *Dsg1a*, *Dsg1b* and *Dsp* promoter activities by transient co-transfection assays in NCTC cells using the previously described reporter vectors [47]. *Dsg1a*, *Dsg1b*, and *Dsp* promoter activities were stimulated by 7.5-, 6.5- and 8.7-fold, 5.8-, 9.9- and 10.8-fold, and 9.6-, 3.5- and 9.6-fold, respectively, when co-transfected with Klf4, Klf5, or both (Fig. 5B). Relative to Klf4, Klf5 had a comparable effect on *Dsg1a*, stronger stimulatory effect on *Dsg1b* and weaker stimulatory effect on *Dsp* promoter activities (Fig. 5B). Co-transfection with both Klf4 and Klf5 did not result in an additive or synergistic stimulation of these promoter activities (Fig. 5B), suggesting that Klf4 and Klf5 function through the same *cis*- elements within the *Dsg1a*, *Dsg1b*, and *Dsp* promoters [47]. Taken together with our previous report [47], these results demonstrate that one of the ways by which Klf4 and Klf5 contribute to corneal epithelial homeostasis is by regulating the expression of desmosomal components Dsg1a, Dsg1b and Dsp.

### Influence of Klf4 and Klf5 on gene regulatory networks in the cornea

In order to determine the influence of Klf4 and Klf5 on gene regulatory networks in the cornea, we examined the expression of other transcription factors in PN11 and PN56 WT and *Klf5*CN corneas and compared them with the previous results from PN56 *Klf4*CN corneas [21]. Comparative analysis of the transcription factors decreased in more than one dataset (i.e., *(a)* PN56 *Klf4*CN vs. WT, *(b)* PN56 *Klf5*CN vs. WT, *(c)* PN11 *Klf5*CN vs. WT, and (d) PN56 WT vs. PN11 WT) identified *Pax6*, *Bnc1*, *Cux1*, *Tox* and *Satb1* as common targets of Klf4 and Klf5 that were also modulated during corneal maturation (Table 12). Pathway analysis of the affected transcription factors revealed distinct networks predominantly associated with development and tissue homeostasis (Figures S2 and S3). The differences in these associated networks in spite of the five common transcription factor targets for Klf4 and Klf5 are consistent with their non-redundant functions in the mouse cornea. Among the common transcription factor targets of Klf4 and Klf5, while Pax6 and Bnc1 are known to regulate corneal epithelial homeostasis [5], [14], [48]–[52], corneal functions of Cux1, Tox and Satb1 are not yet known. Increased expression of Cux1, which suppresses collagen synthesis [53], is consistent with the decreased expression of collagens during WT corneal maturation. The transcription factors whose expression increased in PN56 *Klf4*CN and both PN11 and PN56 *Klf5*CN corneas (*Atf3*, *Litaf*, *Runx1*, *Nfkbie* and *Fhl2*; Table 12) are known to be upregulated during inflammation [54]–[57], raising a possibility that their increased expression reflects pro-inflammatory conditions in these corneas and may not be due to direct de-repression in the absence of Klf4 or Klf5.

**Table 12:** Transcription factors (TFs) differentially expressed in more than one comparison.

Transcription factor	*Klf4*CN vs. WT (PN56)	*Klf5*CN vs. WT (PN56)	*Klf5*CN vs. WT (PN11)	PN56 WT vs. PN11 WT
*Klf5*	1.14	0.028	0.027	2.20
*Bnc1*	0.36	0.45	0.27	(1.58)
*Pax6*	0.38	(0.64)	0.16	12.95
*Cux1*	0.37	(0.60)	0.47	3.56
*Satb1*	0.24	0.43	(0.56)	0.46
*Tox*	0.23	0.29	0.45	0.47
*Sox4*	8.19	4.16	2.10	0.32
*Irf8*	2.38	4.68	2.47	0.46
*Mecom*	1.8	2.30	(1.41)	0.33
*Ar*	1.24	2.85	(1.65)	0.38
*Atf3*	3.77	2.28	3.41	(1.55)
*Fhl2*	2.80	2.98	3.47	(0.77)
*Litaf*	2.04	2.52	2.78	(1.05)
*Nfkbie*	2.22	3.92	3.59	0.41
*Runx1*	2.70	2.05	2.60	2.49

In the first group, *Bnc1*, *Pax6* and *Cux1* have expression profiles most similar to *Klf5*, and therefore are strong candidates for direct control by Klf5 or for co-modulation with Klf5 by an upstream modulator. Members of the second group (*Sox4*, *Irf8*, *Mecom* and *Ar*) have expression profiles which are the inverse of *Klf5*. The third group shows increased expression in PN11 and PN56 *Klf5*CN, and PN56 *Klf4*CN corneas without a convincing change during WT corneal maturation. When the differences were less than 2-fold, those values are shown in parenthesis.

In order to identify if a regulatory relationship exists between different Klfs, we examined the effect of disruption of *Klf5* on expression of other *Klf*s in the cornea. While transcripts for *Klf*s 1, 14 and 15 were essentially absent in the cornea, those for *Klf*s 2, 4, 6, 7, 10, 11, 12, 16 and 17 were present in all samples but showed no robust changes. During WT corneal maturation, the expression of *Klf*s 3, 5 and 6 was increased, while that of *Klf*s 2, 12 and 13 was decreased in PN56 compared with the PN11 corneas. *Klf4* and *Klf5* remained unaffected in *Klf5*CN and *Klf4*CN corneas, respectively, suggesting a lack of regulatory relationship between these two Klfs highly expressed in the cornea. The closely related *Klf9* and *Klf13* [27] were both increased in both *Klf4*CN and *Klf5*CN corneas, suggesting that *Klf9* and *Klf13* show similar compensatory changes whether *Klf5* or *Klf4* is ablated. Whether this represents a true regulatory relationship among Klfs 4, 5, 9 and 13 in the mouse cornea remains to be established.

## Discussion

Previously, we demonstrated that disruption of *Klf5* resulted in defective maturation of the mouse cornea in post-eyelid opening stages [31]. Here, we have employed microarray analysis to obtain a comprehensive view of the changes in corneal gene expression upon deletion of *Klf5* in immature corneas around eyelid opening (PN11) and in adult (PN56) corneas. Our findings reveal the molecular basis of the wide ranging influence of Klf5 on corneal homeostasis and identify candidate target genes of Klf5 in the mouse cornea. In addition, the design of our study allowed us to identify the changes in gene expression between PN11 and PN56 WT corneas.

Earlier reports of Klf5-target gene profiling used chromatin immunoprecipitation followed by microarray (ChIP-Array) in embryonic stem cells [58], or microarray analysis following selective disruption of *Klf5* in the mouse lung [59] or bladder urothelium [60]. Our results are largely consistent with those findings and identify additional Klf5-target genes such as *Pax6* and *Dsg1a*, which play critical roles in the cornea. Differences in Klf5-target genes in the cornea (this study), ESCs [58], lung [59] and bladder urothelium [60] provide evidence for tissue-dependent functions of Klf5. For example, disruption of *Klf5* resulted in increased expression of surfactant protein-D (*Sftpd*) in corneas (Table 2), in contrast to decreased expression in the lung [59], highlighting tissue-dependent functions of Klf5.

A striking feature of our results is the large number of genes whose expression is influenced by the absence of Klf5 in the mouse cornea. By comparison with similar studies of transcription factors such as FoxP2 [61], Sox2 [62], Myb [63] and Bcl11b [64], we predict that Klf5 is likely to directly regulate only a fraction of those genes whose expression is modulated in the *Klf5*CN corneas. The remaining genes are expected to be indirect targets of Klf5, through other transcription factors such as Pax6 [48]–[50], [65], whose expression is reduced in *Klf5*CN corneas (Table 12). Alternatively, they may represent physiological responses to the phenotype brought about by disruption of *Klf5*. For example, a large number of genes upregulated in *Klf5*CN corneas are immune response related, consistent with the massive infiltration of CD45+ cells (Fig. 3), and may not be directly regulated by Klf5. Though it is likely that the *Klf5*CN corneal neovascularization and influx of CD45+ cells indirectly contributed to increased expression of many of the immune response related genes, a similar effect may not be extended to a majority of the negatively regulated genes, which are more likely to represent true targets for Klf5.

Klf4 and Klf5 are both abundantly expressed in the mouse cornea [29], where they play critical, non-redundant roles [22], [31]. In order to understand the molecular basis for their corneal functions, it is necessary to identify and distinguish their target genes. Comparison of the *Klf5*CN corneal gene expression profile (this study) with that of *Klf4*CN corneas [21] revealed that roughly 2/3 of the corneal Klf4- and Klf5-target genes are Klf4- or Klf5-specific, with the rest being common targets. Canonical pathway analysis of genes exclusively modulated by Klf4 yielded “human embryonic stem cell pluripotency” as the most significantly enriched (p<10^−5^) pathway, in agreement with the importance of KLF4 in inducing pluripotency [66]. In contrast, “hepatic fibrosis/hepatic stellate cell activation” was the most significantly enriched (p<10^−12^) pathway among the genes exclusively modulated by Klf5 (Table S10), indicative of a general fibrotic response such as that observed in cultured human keratocytes exposed to TGF-β [67]. Molecular basis for the ability of Klf4 and Klf5 to regulate distinct sets of target genes in spite of possessing identical DNA-binding domain remains to be understood. Genome-wide identification of the nucleotide sequence of Klf4- and Klf5-bound *cis*-elements by chromatin immunoprecipitation followed by large scale sequencing (ChIP-Seq) is necessary to better understand target site selection by Klf4 and Klf5.

Being environmentally exposed, the cornea is frequently exposed to xenobiotic stress. Also, when the eyelids are closed during sleep, the avascular cornea is subjected to almost 75% drop in oxygen partial pressure [68], [69]. Thus, hypoxic and xenobiotic response pathways are expected to play an active role in corneal homeostasis. The important role of inhibitory PAS domain protein (IPAS) - a hypoxia repressor protein- in maintaining corneal avascularity [70], [71] further supports this contention. Moreover, corneal crystallin genes are induced by hypoxia or xenobiotics, further implicating hypoxic and xenobiotic stress in corneal gene expression [16], [72], [73]. Our data demonstrated significant enrichment of xenobiotic metabolism-related pathways among genes whose expression is decreased in *Klf5*CN corneas (Table 11). Thus, we suggest that Klf5 serves an important role in detoxification of the environmentally exposed avascular cornea, by supporting the expression of xenobiotic metabolism related genes.

Important changes take place in the mouse cornea as it matures following eyelid opening around PN12 [8], [74]. This study identified the changes in corneal gene expression between PN11 and PN56, revealing the molecular events underlying post-eyelid opening corneal maturation. Specifically, we demonstrate that during WT corneal maturation between PN11 and PN56, transcripts encoding *(i)* ECM components are sharply decreased, *(ii)* epithelial barrier-related proteins are sharply increased, and *(iii)* members of the solute carrier family proteins are elevated, consistent with *(a)* the active formation of stromal ECM around eyelid opening, with little remodeling taking place in adult corneal stroma, *(b)* rapid stratification of squamous epithelium in post-eyelid opening stages and *(c)* elevated demand for solute transport in the metabolically active adult cornea, respectively.

We compared the present data with a previous analysis of changes in corneal gene expression associated with post-eyelid opening maturation [75], which used Affymetrix MG74Av2 chips targeting a subset (8,666) of the 21,815 unique characterized genes examined here. Applying the present selection rules to their data [75] yielded 442 genes differentially expressed between their immature (PN10) and adult (PN49 to PN56) groups. Though there were differences between the two datasets (which could be attributed to several factors including differences in the mouse strains used, the nature of microarray chips used, and the parameters employed in filtering and analyzing the microarray data), our study confirmed 202 (45.7%) of these genes as differentially expressed between PN11 and PN56 corneas, with 36 concordant increases and 149 concordant decreases (7.0 and 5.1 times the number expected by contingency analysis, respectively, with a χ^2^ test yielding p = 6.8×10^−161^). Thus, the current study confirmed and expanded our knowledge of the changes in gene expression associated with post-eyelid opening corneal maturation [29], [75].

In summary, this report identifies dynamic changes in gene expression accompanying post-eyelid opening corneal maturation, and the role of Klf5 in this process. Our results show that Klf5 contributes to maturation and maintenance of cornea by regulating the expression of subsets of genes involved in specific functions such as cell-cell adhesion, epithelial barrier formation, maintenance of proper level of hydration and xenobiotic metabolism. The changes in *Klf5*CN corneal gene expression are consistent with the elevated immune response and CNV. These results also revealed significant differences between Klf4- and Klf5-target genes, consistent with their non-redundant roles in the mouse cornea. Taken together with our previous report [22], the present studies establish Klf5 as another important node in the genetic network of transcription factors required for corneal maturation and maintenance.

## Materials and Methods

### Ethics Statement

Mice used in these studies were maintained in accordance with the guidelines set forth by the Institutional Animal Care and Use Committee (IACUC) of the University of Pittsburgh, and the ARVO statement on the use of animals in ophthalmic and vision research. All procedures performed on mice reported in this study were approved by the University of Pittsburgh IACUC.

### Conditional disruption of *Klf5*

*Klf5*CN mice were generated on a mixed background by mating *Klf5^loxP/loxP^*, *Le-Cre*/- mice with *Klf5^loxP/loxP^* mice as described before [31]. This mating scheme generated equal numbers of *Klf5^loxP/loxP^*, *Le-Cre/-* (*Klf5*CN) and *Klf5^loxP/loxP^* (control) offspring. Genomic DNA isolated from tail clippings was assayed for the presence of the *Klf5-LoxP* and *Le-Cre* transgenes by PCR using specific primers.

### Isolation of total RNA, quality control, labeling and microarray analysis

WT and *Klf5*CN littermates (4 each at PN56 and 3 each at PN11) were used for comparison of corneal gene expression by microarrays. All corneas used in these studies were microdissected from freshly harvested eyeballs under a surgical microscope. Corneas were dissected using a pair of fine scissors (RS-5611 Vannas Curved Spring Scissors; Roboz Surgical Company, Germany) around the limbus, ensuring that they are free of contamination from iris, ciliary body and/or trabecular meshwork. Two dissected corneas from each mouse were pooled for isolation of total RNA using the RNeasy Mini kit (Qiagen, Germantown, MD). The quality and integrity of the isolated total RNA was confirmed using an Agilent Bioanalyzer (Figure S1); 1.0 µg sample RNAs were subsequently amplified and labeled using a 3′ IVT Express Kit (Affymetrix Inc., Santa Clara, CA) and hybridized to Affymetrix MG 430 2 chips [33]. Utilization of the same amount of total RNA (1.0 µg) from WT and *Klf5*CN corneas for labeling and hybridization ensured that the smaller size of *Klf5*CN corneas did not skew the microarray results.

The raw data obtained from microarray analysis were processed using Affymetrix GeneChip Operating Software (GCOS v 1.4) using software defaults, to assess the presence or absence of each transcript target sequence, its expression level, and then to make all relevant pair-wise statistical comparisons among samples. Expression levels were scaled to a target value of 150 using the software default (2% trimmed mean). Prior to scaling, mean microarray expression levels were 449±38 (mean ± SD, n = 13 with one outlier of 255), and target sequences were detected (called Present) for 57.8%±2.5% (mean ± SD, n = 14) of panels on the chip. After scaling, three redundant panels for the housekeeping gene GAPDH reported coefficients of variation of 12.3%, 10.9% and 13.1% respectively (n = 13 for each panel since each had one outlier value).

Processed data were sorted and inspected in an Excel spreadsheet (Microsoft, Redmond, WA) with BRB-ArrayTools (www.linus.nci.nih.gov/BRB-ArrayTools.html). Genes were considered differentially expressed between any two groups if they satisfied the following four criteria: (1) the average value for the high-expression group was >2 fold greater than the average value for the low expression group; (2) the high-expression group contained at least 3 detectable transcripts (called Present); (3) the differences between groups were significant at p≤0.057 in a two-tailed Mann-Whitney rank sum test. As this requirement could not be met in the PN11 WT vs. PN11 *Klf5*CN comparison, we required the minimum p value possible (0.1) instead; (4) for groups of *m* and *n* members, where *m*≥*n*, of the (*m*×*n*) pair-wise comparisons made by the GCOS software, at least ((*m* - 1)×*n*) show valid differences. We re-analyzed the previously published *Klf4*CN [21] data using current filters to compare PN56 WT corneas with *Klf4*CN corneas, at a cutoff of p = 0.063 in the Mann-Whitney test. MIAME-compliant microarray data were submitted to NCBI GEO (http://www.ncbi.nlm.nih.gov/geo/; Accession Number GSE36229).

### Validation of microarray results by real time QPCR

Applied Biosystems (ABI: Foster City, CA) was the source of the reagents, equipment and software for quantitative real time RT-PCR assays (QPCR). Total RNA isolated from pooled corneas of 10 WT or *Klf5*CN mice was quantified and the concentration adjusted with RNase-free water to 100 ng/µl. cDNA was generated using an ABI High Capacity cDNA Archive Kit and real-time QPCR assays were performed in a ABI StepOnePlus thermocycler using GAPDH as endogenous control for SYBR green assays and Pcx (pyruvate carboxylase) as endogenous control for FAM assays; the results were analyzed using ABI StepOnePlus software. Nucleotide sequence of different primers used is given in Table S1.

### Immunofluorescent staining of corneal whole mounts

Corneas were dissected, flattened by three radial incisions, washed 3 times for 15 minutes each in PBS with 4% FBS and blocked in FC block (BD Pharmingen, San Jose, CA) for 20 minutes prior to incubation with FITC-conjugated anti-CD45 antibody, or FITC-conjugated anti-Lyve1 antibody and PE-conjugated anti-CD31 antibody (BD Pharmingen) overnight at 4°C. Corneas were then washed three times each for 30 minutes in PBS/4% FBS, fixed in 1% Paraformaldehyde for 2 hours at 4°C, rinsed 3 times again for 30 minutes each in PBS/4% FBS and mounted in Aqua Poly/Mount (Polysciences Inc, Warrington, PA). Images were acquired on a Nikon Ti-E and/or Olympus Fluoview 1000 confocal system with an Olympus IX81 microscope. Stacks were imaged at 20× (Numerical Aperture 0.85) and 60× (Numerical Aperture 1.42) through the cornea. Individual images were stitched together using Metamorph (Molecular Devices, Sunnyvale, CA) and Photoshop (Adobe Systems Inc., San Jose, CA) programs.

### Immunofluorescence

Paraformaldehyde fixed, paraffin-embedded sections were deparaffinized with xylene, blocked with 10% goat serum in PBS for 1 h at room temperature (RT) in a humidified chamber, washed twice with PBS for 5 minutes each, incubated overnight with a 1∶50 dilution of anti-Dsg (recognizes both Dsg1a and Dsg1b) or anti-Dsp primary antibody raised in rabbit (Santa Cruz Biotechnology, Santa Cruz, CA) at 4°C, washed thrice with PBS for 5 minutes each, incubated with secondary antibody (AlexaFluor 546-coupled goat anti-rabbit IgG antibody; Molecular Probes, Carlsbad, CA) at a 1∶300 dilution for 1 h at RT, washed twice with PBS for 5 minutes each, incubated with DAPI for 10 minutes, mounted with Aqua Polymount (Polysciences, Inc), and observed with a fluorescence microscope. All images presented within each composite figure were acquired under identical settings and processed in a similar manner using Adobe Photoshop and Illustrator (Adobe, Mountain View, CA).

### Reporter Vectors, Cell Culture, and Transient Transfection Assays

Different reporter vectors where approximately 2 kb *Dsg1a*, *Dsg1b* and *Dsp* promoter fragments drive the expression of luciferase reporter gene were described earlier [47]. Full-length *Klf4* in pCI-*Klf4* and full length *Klf5 in pCMV-Sport-Klf5* was transiently expressed using the CMV promoter. Human skin keratinocyte NCTC cells were grown in Dulbecco's modified Eagle's medium(DMEM) supplemented with 10% fetal bovine serum, penicillin, and streptomycin at 37°C in a humidified chamber containing 5% CO_2_ in air. NCTC cells in 12-well plates were co-transfected with 0.5 µg *pDsg1a-Luc*, *pDsg1b-Luc* or *pDsp-Luc*, along with 20 ng *pRL-SV40* (for normalization of transfection efficiency) and 0.5 µg *pCI* (control), or 0.25 µg each of *pCI* and pCI-*Klf4*, or *pCI* and *pCMV-Sport6-Klf5*, or pCI-*Klf4* and *pCMV-Sport6-Klf5* using 2.5 µL transfection reagent (Expressfect; Denville Scientific). After 2 days, cells were washed with PBS and lysed with 200 µL passive lysis buffer (Promega). 50 µL of lysate was analyzed using a dual-luciferase assay kit (Promega) and a microplate luminometer (Synergy-II; Biotek Instruments, Winooski, VT). The measurement was integrated over 10 seconds, with a delay of 2 seconds. Firefly luciferase activity normalized for transfection efficiency using the SV40 promoter-driven *Renilla* luciferase activity, were used to obtain mean promoter activities. Fold-activation was determined by dividing mean promoter activity by the promoter activity with only pCI. Error bars indicate Standard Error of Mean (SEM). Results presented are representative of three independent experiments.

## Supporting Information

Figure S1 **Quality of RNA used for microarray analysis.** Total RNA isolated from PN11 and PN56 WT or *Klf5*CN corneas was subjected to Agilent Bioanalyzer analysis using nanoRNA chips. Resultant gel image confirming the RNA integrity is shown. Corresponding RNA Integrity Value (RIN) numbers are provided at the top of each lane.
(TIF)

Figure S2 **Comparative analysis of the network of target genes influenced by transcription factors that are affected in** ***Klf4*** **CN corneas.** Lists of transcription factors downregulated by 1.5-fold in *Klf4*CN corneas (shaded yellow) were submitted to Ingenuity Pathway Analysis (IPA), where gene identifiers were mapped to their corresponding gene objects and overlaid onto a global molecular network in the Ingenuity Pathways Knowledge Base to generate the associated networks. Direct relationships are shown with solid arrows and indirect relationships with dashed arrows. Legends for different shapes used are shown.
(TIF)

Figure S3 **Comparative analysis of the network of target genes influenced by transcription factors that are affected in** ***Klf5*** **CN corneas.** Lists of transcription factors downregulated by 1.5-fold in *Klf5*CN corneas (shaded yellow) were submitted to Ingenuity Pathway Analysis (IPA), where gene identifiers were mapped to their corresponding gene objects and overlaid onto a global molecular network in the Ingenuity Pathways Knowledge Base to generate the associated networks. Direct relationships are shown with solid arrows and indirect relationships with dashed arrows. Legends for different shapes used are shown.
(TIF)

Table S1 **Sequence of nucleotide primers used for QPCR.**
(XLSX)

Table S2 **Most downregulated genes in PN11** ***Klf5*** **CN corneas.**
(XLS)

Table S3 **Most upregulated genes in PN11** ***Klf5*** **CN corneas.**
(XLS)

Table S4 **Most downregulated genes in PN56** ***Klf5*** **CN corneas.**
(XLS)

Table S5 **Most upregulated genes in PN56** ***Klf5*** **CN corneas.**
(XLS)

Table S6 **Most upregulated genes in PN56 WT compared with PN11 WT corneas.**
(XLS)

Table S7 **Most downregulated genes in PN56 compared with PN11 WT corneas.**
(XLS)

Table S8 **Genes whose expression is decreased at PN56 in both** ***Klf4*** **CN and** ***Klf5*** **CN corneas.** Transcription factors are in **bold**. Genes are annotated by other effects as follows: (*) = developmental effect, (**) = PN11 *Klf5*CN effect, (***) = both. All 43 developmental effects are increases except Agpat3, Lamb1-1, Ntn1, Satb1 and Tox. All 48 PN56 *Klf5*CN effects are decreases except Vgll3.
(XLSX)

Table S9 **Genes whose expression is increased in both** ***Klf4*** **CN and** ***Klf5*** **CN corneas at PN56.** Transcription factors are highlighted in bold. Genes are annotated by the other effects. (*) = developmental effect, (**) = *Klf5*CN effect in PN11 cornea also, (***) = both. All changes in the PN11 Klf5CN are increases and all developmental changes are decreases, except those shaded grey.
(XLSX)

Table S10 **Canonical pathways enriched in PN11 and PN56 corneal Klf5-target genes or in developmentally-modulated genes.** Aggregates of differentially expressed genes in PN11or PN56 *Klf5*CN corneas, and during WT corneal maturation were subjected to canonical pathway analysis. Pathways for which at least one group showed significant (−log(p) >5) enrichment were selected. Where upregulated genes exceed downregulated genes by >2-fold, cells are colored red; where downregulated genes exceed upregulated genes by >2-fold, cells are colored green. Otherwise cells are colored yellow. For *Klf5*CN groups, significantly enriched pathways are dominated by upregulated genes. Developmentally modulated pathways contain a fair balance of up- and down-regulated genes.
(XLSX)

Table S11 **Immune-related genes whose expression changes in** ***Klf5*** **CN corneas.** 107 of 368 immun/inflam genes were upregulated in PN56 and/or PN11 *Klf5*CN corneas, with only 7 genes downregulated. Entries in bold show the smaller number of genes affected in PN56 *Klf4*CN corneas.
(XLSX)
