# Supplementary figures and images for: Critical Role of Klf5 in Regulating Gene Expression during Post-Eyelid Opening Maturation of Mouse Corneas
Source: PLoS One. 2012 Sep 14;7(9):e44771. doi: 10.1371/journal.pone.0044771 (PMC3443110; doi:10.1371/journal.pone.0044771)

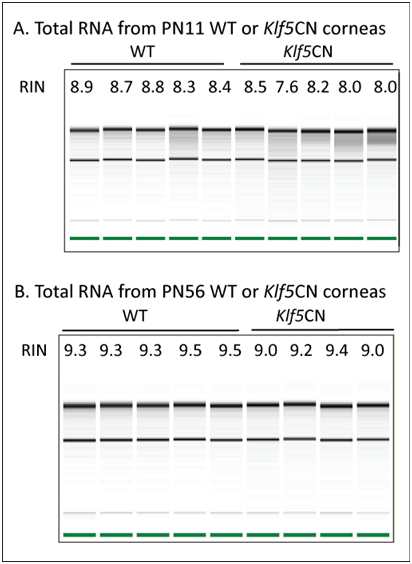

Supplement: Figure S1 — Quality of RNA used for microarray analysis. Total RNA isolated from PN11 and PN56 WT or Klf5CN corneas was subjected to Agilent Bioanalyzer analysis using nanoRNA chips. Resultant gel image confirming the RNA integrity is shown. Corresponding RNA Integrity Value (RIN) numbers are provided at the top of each lane. (TIF) [file pone.0044771.s001.tif]

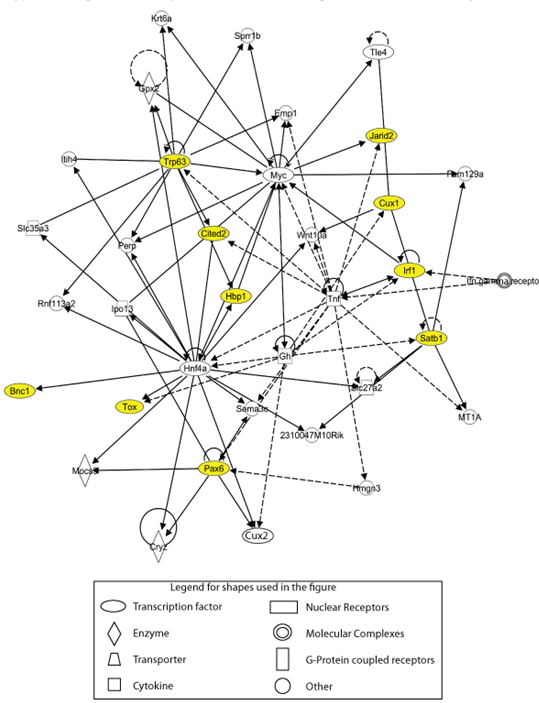

Supplement: Figure S2 — Comparative analysis of the network of target genes influenced by transcription factors that are affected in Klf4 CN corneas. Lists of transcription factors downregulated by 1.5-fold in Klf4CN corneas (shaded yellow) were submitted to Ingenuity Pathway Analysis (IPA), where gene identifiers were mapped to their corresponding gene objects and overlaid onto a global molecular network in the Ingenuity Pathways Knowledge Base to generate the associated networks. Direct relationships are shown with solid arrows and indirect relationships with dashed arrows. Legends for different shapes used are shown. (TIF) [file pone.0044771.s002.tif]

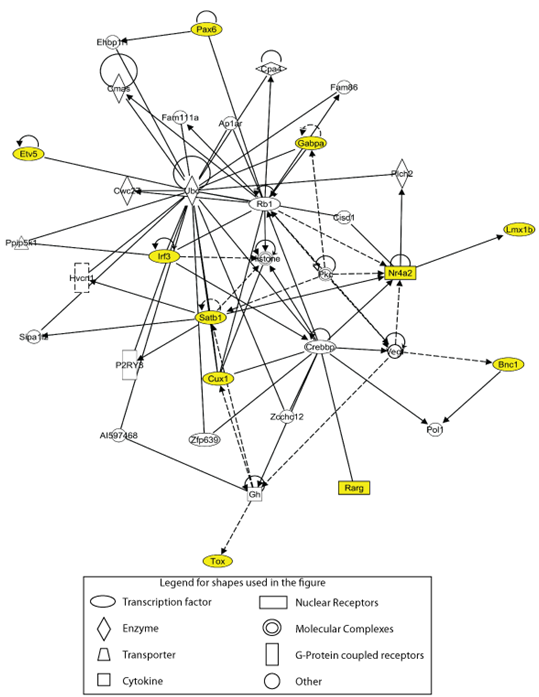

Supplement: Figure S3 — Comparative analysis of the network of target genes influenced by transcription factors that are affected in Klf5 CN corneas. Lists of transcription factors downregulated by 1.5-fold in Klf5CN corneas (shaded yellow) were submitted to Ingenuity Pathway Analysis (IPA), where gene identifiers were mapped to their corresponding gene objects and overlaid onto a global molecular network in the Ingenuity Pathways Knowledge Base to generate the associated networks. Direct relationships are shown with solid arrows and indirect relationships with dashed arrows. Legends for different shapes used are shown. (TIF) [file pone.0044771.s003.tif]
